# Janus Nanostructures Enabled by Confined Interfacial Engineering for Enhanced Electromagnetic Response

**DOI:** 10.1002/advs.77021

**Published:** 2026-08-06

**Authors:** Limeng Song, Cheng Song, Feiyue Hu, Yongqiang Chen, Linan Wang, Huagao Wang, Li Guan, Duo Chen, Kesong Yu, Yabo Huang, Shihao Jia, Xin Xu, Qiancheng Gao, Dou Zhang, Hailong Wang, Haijiao Xie, Rui Zhang, Bingbing Fan

**Affiliations:** ^1^ School of Materials Science and Engineering Zhengzhou University of Aeronautics Zhengzhou P. R. China; ^2^ School of Materials Science and Engineering Zhengzhou University Zhengzhou P. R. China; ^3^ State Key Laboratory of Engineering Materials For Major Infrastructure School of Materials Science and Engineering Southeast University Nanjing P. R. China; ^4^ Institute of Advanced Ceramics Henan Academy of Sciences Zhengzhou P. R. China; ^5^ Department of Astronautical Science and Mechanics Harbin Institute of Technology Harbin P. R. China; ^6^ Hangzhou Yanqu Information Technology Co., Ltd Hangzhou P. R. China

**Keywords:** confined interfacial engineering, electromagnetic wave absorption, impedance matching regulation, janus nanostructures, SiC@C hollow nanoshells

## Abstract

Regulating electromagnetic responses through interfacial design has attracted increasing attention, while the precise construction and mechanism understanding of asymmetric nano‐interfaces remain largely unexplored, particularly at the nanoscale. Herein, taking a representative carbon‐based microwave absorber as a model system, we develop a confined diffusion‐mediated interfacial engineering strategy to construct SiC@C Janus hollow nanostructures with well‐defined asymmetric nano‐interfaces, enabling the coordinated regulation of interfacial configuration and electromagnetic functionality. At an ultralow filler loading of 10 wt.%, the optimized composite achieves a minimum reflection loss of ‐62.36 dB, an effective absorption bandwidth of 7.82 GHz, together with a pronounced radar cross section reduction. Combined theoretical analysis, electromagnetic simulations, and multiscale characterizations reveal that the asymmetric Janus nano‐interfaces promote improved impedance matching via refined dielectric modulation. Meanwhile, the coupling of the hollow architecture with heterogeneous interfaces induces multiple reflections, scattering, and interfacial polarization, thereby synergistically enhancing electromagnetic energy dissipation. This work provides nanoscale insights into the electromagnetic modulation mechanism governed by asymmetric Janus interfaces and establishes a general paradigm of confined interfacial engineering, offering new theoretical guidance and structural design strategies for next‐generation high‐performance electromagnetic protection materials.

## Graphical abstract

Confined diffusion‐driven interfacial engineering enables the nanoscale construction of asymmetric SiC@C Janus hollow nanoshells, achieving synergistic impedance matching and electromagnetic energy dissipation for highly efficient broadband microwave absorption at ultralow filler loading.

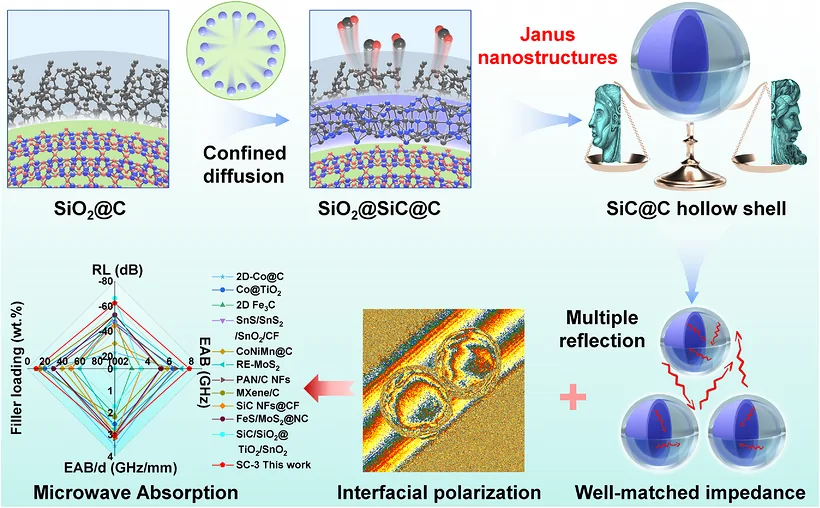

## 1 Introduction

The rapid advancement of wireless communication, radar detection, and electronic information technologies has continuously expanded the spectral range, intensity, and application scenarios of electromagnetic waves, leading to an increasingly complex electromagnetic environment [1, 2, 3]. Accordingly, electromagnetic interference (EMI) and electromagnetic pollution pose potential risks to information transmission security, the reliable operation of precision electronic devices, and human health [4, 5, 6]. Given the increasing demand for electromagnetic protection, research on novel functional materials capable of efficient electromagnetic wave absorption (EMA) has become a major focus in materials science and electromagnetic protection [7]. Ideal EMA materials are required not only to realize significant attenuation of electromagnetic energy over a broad frequency range, but also to possess lightweight characteristics, reduced thickness, and structural designability in order to meet the urgent demands of aerospace, national defense equipment, and next‐generation communication systems [8, 9]. However, existing EMA material systems often struggle to achieve the synergistic optimization of impedance matching and electromagnetic attenuation capability, and this intrinsic contradiction has become a central bottleneck restricting further performance enhancement [10, 11].

In recent years, structural design has been regarded as an effective strategy to overcome the above limitation [12]. Among various approaches, asymmetric interfacial structures, known as Janus structures, provide new degrees of freedom for regulating electromagnetic responses due to their asymmetric spatial composition and functional distribution [13]. Previous studies have demonstrated that Janus structures exhibit unique advantages in multiphysical field regulation, energy transport, and interfacial coupling, and they also show potential application value in the field of electromagnetic protection [14]. Nevertheless, current investigations of Janus structures are mainly focused on macroscopic layered systems. For example, Jiang et al. [15]. reported a novel porous cellulose acetate (PCA) /Ag Janus membrane prepared through the combined effect of cellulose acetate phase separation and a self‐stratification process driven by high‐density silver fillers. Owing to the opposite optical properties of the PCA and Ag layers, together with the high thermal conductivity and excellent electrical conductivity of the Ag layer, the membrane can rapidly diffuse radiative cooling or heating effects to the underlying space while achieving an EMI shielding efficiency of 70 dB. Feng et al. [16]. developed a Janus fabric that integrates radiative cooling, solar heating, and EMI shielding through a uniformly distributed bilayer structure. The radiative cooling layer consists of boron nitride nanosheets embedded in porous polyurethane, whereas the solar heating and EMI shielding layer is composed of MXene‐doped porous polyurethane. Overall, prevailing design strategies primarily rely on stacking distinct functional layers or spatial partitioning, while the intrinsic role of interfacial structures in electromagnetic response regulation remains insufficiently explored. Compared with macroscopic configurations, nanoscale interfaces exhibit higher volumetric polarization density and enhanced electromagnetic dissipation capability. Nevertheless, the controllable construction of asymmetric Janus nano‐interfaces and the fundamental mechanisms underlying their regulation of impedance matching and electromagnetic attenuation remain largely unexplored. This knowledge gap has hindered the rational design and further advancement of Janus architectures toward high‐efficiency EMA applications [17].

From a structural design perspective, the effective realization of Janus nanostructures generally relies on functional hosts that simultaneously provide abundant interfacial coupling sites and high structural designability. Carbon‐based materials, featuring high specific surface area, tunable dielectric response, and flexible microstructural regulation capability, are therefore regarded as ideal platforms for constructing nanoscale Janus interfaces and are widely considered promising candidates for next‐generation high‐performance EMA materials [18]. However, conventional carbon‐based EMA materials predominantly depend on conduction loss to achieve electromagnetic attenuation, which is often accompanied by impedance mismatch that enhances electromagnetic wave reflection and thus reduces overall absorption efficiency [19]. To address this limitation, multiphase heterogeneous interfaces have been introduced to generate interfacial polarization and charge accumulation, leading to partial enhancement of electromagnetic attenuation [20]. Nevertheless, existing strategies largely rely on the superposition of interfacial quantity or random multiphase distribution, lacking intrinsic control over interfacial morphology and scale, which can elevate the effective permittivity and consequently deteriorate impedance matching. Meanwhile, the electromagnetic response of interfaces remains difficult to regulate at the nanoscale, resulting in insufficiently precise control of electromagnetic energy dissipation and thereby constraining further improvements in broadband stability and high‐intensity absorption [21]. In contrast, the inherent spatial asymmetry of Janus interfaces in composition and local electromagnetic parameter distribution offers a new structural pathway for the simultaneous regulation of incident electromagnetic coupling and energy dissipation [22]. Realizing nanoscale construction of Janus interfaces in carbon‐based systems, while elucidating the intrinsic relationship between interfacial structure and electromagnetic response, is essential to overcoming the performance limits of conventional carbon‐based absorbers. Accordingly, developing precise fabrication strategies for nanoscale Janus architectures and clarifying their electromagnetic regulation mechanisms remains a central scientific challenge in the field of electromagnetic protection materials [23].

Based on the above considerations, this study employs widely used carbon‐based absorbers as the research platform and proposes an interfacial engineering strategy based on confined diffusion to construct SiC@C Janus hollow nanospheres with asymmetric structural characteristics at the nanoscale. The Janus nano‐interface is controllably formed through in situ reactions within a confined interfacial region, enabling excellent EMA performance under low filler loading. By integrating theoretical calculations, electromagnetic simulations, and multiscale characterization techniques, the critical mechanisms by which asymmetric hollow Janus nano‐interfaces regulate dielectric response, improve impedance matching, and enhance electromagnetic energy dissipation are systematically elucidated. This work expands the structural connotation and functional understanding of Janus asymmetric interfaces in EMA materials at the nanoscale and proposes a universal confined interfacial engineering concept, thereby providing new theoretical guidance for the structural design of high‐efficiency and designable electromagnetic protection materials, with broad application prospects in aerospace and communication electronics.

## 2 Results and Discussion

### 2.1 Microstructure, Phase, and Chemical Composition of the Janus Nanostructures

Figure 1a shows the schematic illustration of the synthesis of the SiC@C Janus hollow nanospheres. First, SiO_2_ nanospheres were obtained via the hydrolysis of tetraethyl orthosilicate (TEOS), followed by coating with phenolic resin to form SiO_2_@phenolic resin, which was subsequently carbonized to yield SiO_2_@C. Upon further increasing the temperature to 1400°C, carbothermal reduction reactions were induced at the C/SiO_2_ interface (SiO_2_ + 3C → SiC + 2CO↑ and SiO_2_ + 4CO → SiC + 3CO_2_↑), enabling the in situ formation of SiC [24]. Meanwhile, tuning the amount of phenolic resin precursor regulates the growth of the outer carbon layer and restricts atomic diffusion during the reaction process, leading to the formation of a SiO2@SiC@C sandwich‐like intermediate structure. Subsequent HF etching produces SiC@C Janus hollow nanospheres with asymmetric structural characteristics. By progressively increasing the phenolic resin content, the resulting samples are denoted as SC‐1 to SC‐5, enabling the continuous regulation of particle size and interfacial structural evolution. For comparison, carbon‐shell‐only and SiC‐shell‐only counterparts prepared under identical conditions are designated as CS and SS, respectively. Detailed experimental parameters are provided in the Experimental Section. Figure S1 presents the x‐ray diffraction (XRD) patterns of the samples. The CS and SC series exhibit a broad hump between 20° and 30°, indicating the amorphous nature of the carbon layer. The main diffraction peaks of the SS and SC series match well with the standard card of 3C‐SiC (PDF#29‐1129) [25]. The peaks located at 35.6°, 60.0°, and 71.8° correspond to the (111), (220), and (311) crystal planes of β‐SiC, respectively, confirming the presence of cubic SiC in the samples and demonstrating the successful fabrication of SiC/C composites via the confined interfacial engineering strategy.

**FIGURE 1:**
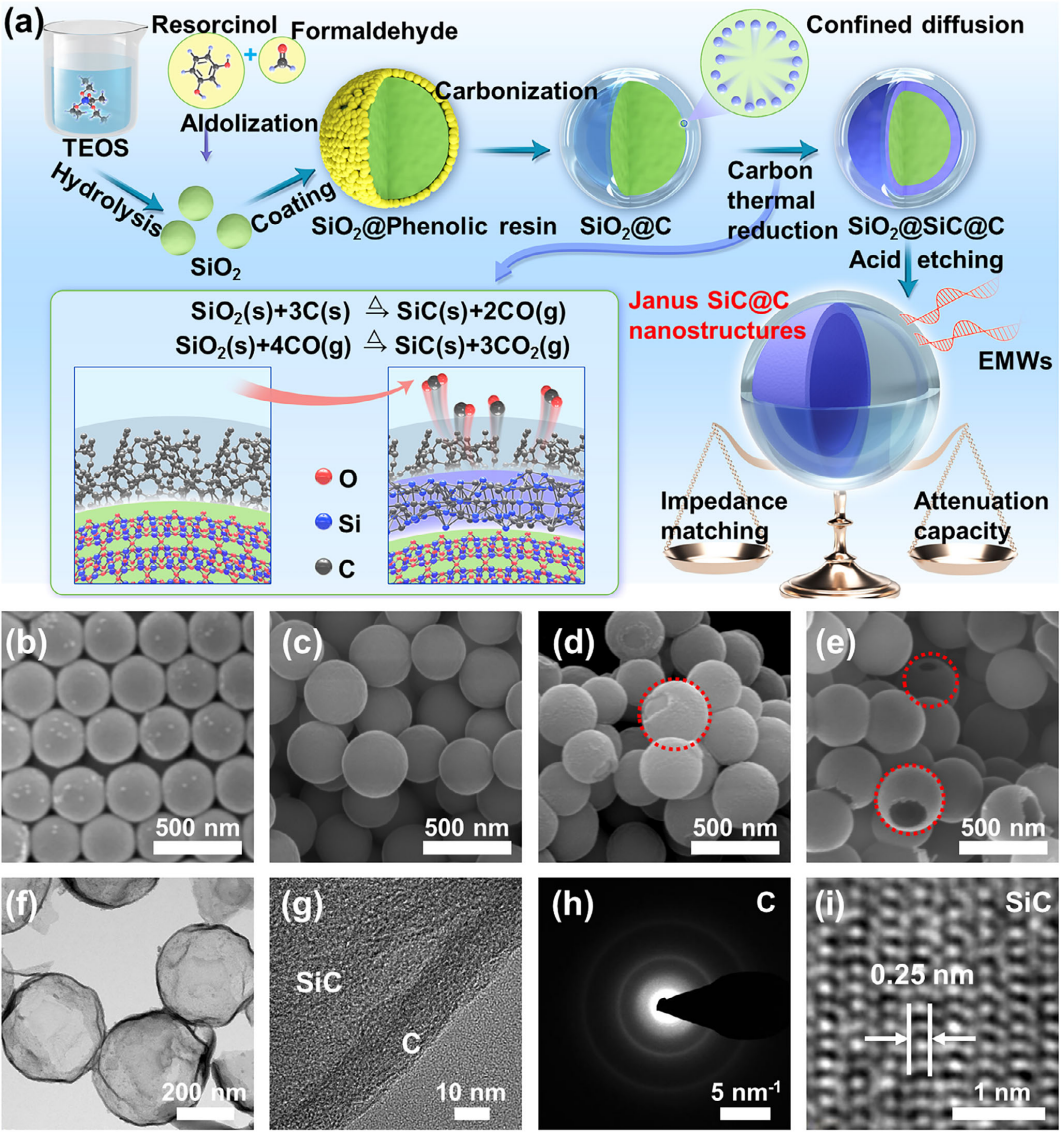
(a) Schematic illustration of the preparation process of Janus SiC@C hollow nanospheres; SEM images: (b) SiO_2_ nanospheres, (c) SiO_2_@phenolic resin‐3, (d) SiO_2_@SiC@C‐3, and (e) SC‐3 sample; (f) TEM images and (g‐i) HRTEM images of the SC‐3 sample, where (h) is the carbon layer (in SAED pattern) and (i) is the SiC layer (at a higher magnification in HRTEM).

In addition, the Fourier transform infrared (FTIR) spectra in Figure S2 display a characteristic Si─C stretching vibration at 803 cm^−1^ for both SS and SC samples. Compared with SS, the CS and SC series show additional absorption bands at 1114 and 475 cm^−1^, corresponding to the C─O─C ether linkage from carbonized residual phenolic resin and the C─C stretching of the aromatic carbon framework, respectively, further confirming the successful formation of SiC/C composite structures. Raman spectra (Figure S3) reveal that, relative to the CS sample, the SS and SC samples exhibit an additional characteristic peak at 795 cm^−1^, associated with the transverse optical (TO) mode of Si─C vibration at the Γ point [26]. Meanwhile, the characteristic peaks at approximately 1343 and 1598 cm^−1^ for all samples correspond to the D band of amorphous carbon and the G band of graphitic carbon, respectively [27]. The x‐ray photoelectron spectroscopy (XPS) survey spectrum in Figure S4a indicates that the SC‐3 sample mainly consists of Si, C, and O elements. High‐resolution Si 2p spectra (Figure S4b) reveal two peaks at binding energies of 100.9 and 103.1 eV, corresponding to Si─C and Si─O bonds, respectively. The C 1s spectrum (Figure S4c) can be deconvoluted into two components located at 282.9 eV (C─Si) and 284.8 eV (C─C) [28]. In the O 1s spectrum (Figure S4d), a characteristic peak at 532.3 eV is observed, which is attributed to the O─Si bond.

Figure 1b shows the SiO_2_ nanospheres obtained via TEOS hydrolysis, which exhibit a regular spherical morphology with an average diameter of approximately 200 nm. Figure 1c and Figures S5 and S6 present SiO_2_@phenolic resin structures with different coating thicknesses, indicating that the particle size gradually increases with increasing coating amount, reaching nearly 350 nm at the maximum thickness. As shown in Figure 1d, the SiO_2_@SiC@C material displays a distinct core‐shell layered morphology with notches formed under high‐temperature carbothermal reduction. The particle size remains essentially unchanged compared with that before heat treatment, suggesting that the coating layer provides an effective interfacial confinement effect, enabling the synthesis of Janus nanostructures. Figure 1e shows the SEM image of SiC@C core‐shell hollow nanospheres obtained after HF etching of SiO_2_@SiC@C. The particles exhibit a uniform spherical morphology with smooth surfaces and visible openings, which facilitate air penetration for impedance matching regulation and promote multiple internal scattering of electromagnetic waves. Meanwhile, the morphologies of CS and SS in Figures S7 and S8 are similar to that of the SC samples.

The transmission electron microscopy (TEM) images in Figure 1f reveal that SC‐3 possesses a hollow core‐shell structure. Further energy‐dispersive x‐ray spectroscopy (EDS) elemental mapping (Figure S9) shows that the outer layer is enriched in carbon, while Si and C are uniformly overlapped in the inner layer, confirming that SC‐3 consists of an outer carbon shell and an inner SiC layer. A small amount of oxygen is also detected, consistent with the XPS results, indicating slight surface oxidation. The high‐resolution TEM (HRTEM) images and selected‐area electron diffraction (SAED) patterns of SC‐3 shown in Figure 1g–i and S10 reveal an amorphous carbon shell, while a lattice spacing of 0.25 nm, corresponding to the (111) plane of SiC, is clearly observed, confirming the crystallinity of the SiC layer. Together, these morphological characterizations validate the successful formation of SiC@C Janus hollow nanospheres and highlight the reproducibility and versatility of the confined diffusion interfacial engineering strategy.

### 2.2 EMA Properties of the Janus Nanostructures

The scattering parameters (S‐parameters) of the hollow nanoshell samples with a filling ratio of 10 wt.% were measured in the frequency range of 2–18 GHz using a vector network analyzer (VNA). The electromagnetic parameters, including complex permittivity [ε_r_ = ε׳−jε״] and permeability [µ_r_ = µ׳−jµ״], were obtained from the S‐parameters based on the Nicolson–Ross–Weir (NRW) method [29]. The real parts ε′ and µ′ represent the electromagnetic energy storage capability, whereas the imaginary parts ε″ and µ″ determine the dielectric and magnetic attenuation behavior of the materials [30]. Subsequently, the reflection loss (RL) of the hollow nanoshells was calculated from the electromagnetic parameters using transmission line theory and employed as the evaluation criterion for EMA performance, with the corresponding equations provided in the Experimental Section. When RL ≤ −10 dB, more than 90% of incident electromagnetic waves are absorbed, and the corresponding frequency range is defined as the effective absorption bandwidth (EAB) [31]. Figure 2a–c present the 3D RL distributions of CS, SC‐3, and SS. Compared with CS, the incorporation of SiC significantly enhances electromagnetic attenuation, with the minimum reflection loss (RL_min_) improving from −13.79 to −62.36 dB and achieving over 99.9999% absorption at a thickness of 2.54 mm. Although SS exhibits an RL_min_ of −15.98 dB, the corresponding matching thickness increases to 4.99 mm, indicating that the intrinsic attenuation capability of pure SiC hollow nanoshells remains limited. From the 2D RL contour maps in Figure 2d–f, SC‐3 exhibits the largest EAB of 7.82 GHz among CS, SC‐3, and SS. Together with Figures S11–S15 and Figure 2g, SC‐3 demonstrates the optimal combination of RL_min_ and EAB across all prepared hollow nanoshells. Importantly, this superior performance is not an isolated result but is consistent with the systematic evolution observed in SC‐1 to SC‐5, where precursor‐regulated structural variation leads to continuous changes in particle morphology and corresponding electromagnetic response, thereby establishing a coherent structure–property correlation and supporting the controllable fabrication of SiC@C Janus hollow architectures. Furthermore, the RL_min_ and EAB of SC‐3 at different coating thicknesses (Figure 2h) confirm its strong absorption intensity and broadband response over a wide thickness range. Therefore, its EMA performance was comprehensively compared with recently reported advanced absorbers, including carbon‐based, SiC‐based, and magnetic absorbers (Figure 2i) [24, 32, 33, 34, 35, 36, 37, 38, 39, 40, 41]. Remarkably, SC‐3 achieves excellent absorption even at an ultralow filler loading of 10 wt.% due to the synergistic effects of its hollow structure, low‐permittivity SiC core, and Janus heterointerfaces, which collectively enhance impedance matching, provide multiple reflection/scattering paths, and generate abundant polarization centers, as summarized in Table S1. These features enable SC‐3 to deliver strong attenuation, broad EAB, and superior thin‐layer performance, highlighting its advantages in a unified evaluation framework considering RL_min_, EAB, and the corresponding matching thickness of EAB (d_2_).

**FIGURE 2:**
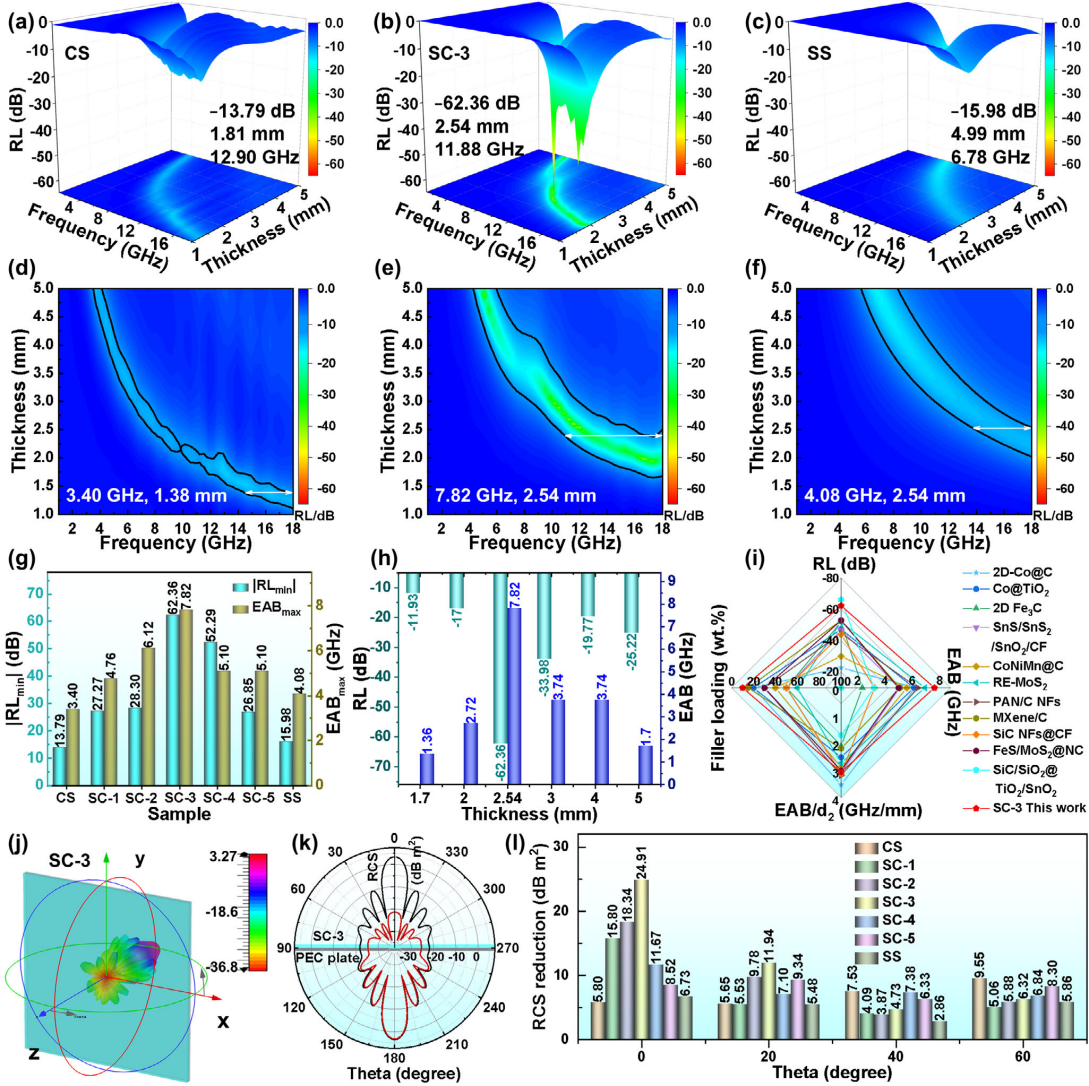
3D RL plots of (a) CS, (b) SC‐3, and (c) SS; 2D representations of RL for (d) CS, (e) SC‐3, and (f) SS; (g) Comparison of |RL_min_| and EAB_max_ for the samples; (h) RL_min_ and EAB of the SC‐3 sample at different thicknesses; (i) Performance comparison between SC‐3 and other advanced microwave absorbers; (j) 3D RCS plot of the PEC coated with the SC‐3 absorber layer; (k) Polar RCS plot of the PEC coated with the SC‐3 layer; (l) RCS reduction values of all samples.

Furthermore, CS, SS, and SC series materials were employed as coatings to evaluate their stealth performance in protecting a perfect electric conductor (PEC) plate. Consistent with the metal‐backed model in transmission line theory, CST Studio Suite was used to simulate the radar cross‐section (RCS) reduction of EMA coatings under far‐field conditions for representative targets such as aircraft and vessels [42]. Figures S16 and S17 and Figure 2j present the RCS results of the bare PEC plate and the PEC plate coated with hollow nanoshell absorbers, showing that the bare PEC exhibits the strongest scattering signal, whereas all nanoshell‐coated samples display significantly reduced 3D radar scattering intensities. Figure S18 and Figure 2k further demonstrate that the SC‐3 coating delivers the most effective stealth performance, exhibiting the lowest RCS values and remaining below −20 dB m^2^ within an angular range of ‐60° to 60°, highlighting the superiority of the Janus interfacial design. In addition, the RCS reduction value, defined as the difference between the RCS of the bare PEC plate and that of the absorber‐coated PEC plate, serves as a key stealth metric. As shown in Figure 2l, SC‐3 achieves the maximum RCS reduction of 24.91 dB m^2^ at θ = 0°, exceeding the typical requirement of 10 dB m^2^ for modern stealth aircraft [43]. These results indicate that the Janus nanostructured material holds significant practical potential as a new class of electromagnetic protection and stealth material.

Notably, the SiC@C Janus hollow nanospheres integrate outstanding microwave absorption capability with favorable structural stability, which is essential for practical electromagnetic protection applications. The thermochemically stable SiC framework provides excellent oxidation resistance and structural robustness, while the strongly coupled SiC/C heterointerfaces help maintain interfacial integrity and preserve the synergistic electromagnetic response under practical service conditions. Moreover, the carbon shell not only contributes to conductive loss and interfacial polarization but also serves as a protective layer for the inner SiC component, further improving structural durability. Benefiting from their nanoscale spherical morphology and hierarchical Janus architecture, these nanostructures are expected to exhibit good compatibility with polymer matrices and provide potential for integration into lightweight EMA coatings.

### 2.3 EMA Mechanism of the Janus Nanostructures

To elucidate the intrinsic origin of the enhanced EMA performance of the Janus hollow nanoshells, their microwave loss characteristics were systematically investigated. First‐principles calculations were employed to determine the work functions of the constituent components, as shown in Figure S19 and Figure 3a. The results indicate that the work function of the carbon layer (5.000 eV) is lower than that of SiC (5.636 eV). On this basis, an interface model analogous to a metal/semiconductor junction is constructed. Owing to this work function difference, electron transfer occurs from the carbon side to the SiC side at the Janus interface, resulting in the formation of a built‐in electric field. Figure 3a schematically illustrates this interfacial charge redistribution process. Differential charge density analyses (Figure 3b,c and Figure S20) demonstrate pronounced charge separation at the SiC/C interface, characterized by charge depletion in the carbon layer and charge accumulation in the SiC region. Under an alternating electromagnetic field, periodic carrier migration and accumulation give rise to space charge displacement polarization, namely interfacial polarization, thereby effectively dissipating incident microwave energy [33].

**FIGURE 3:**
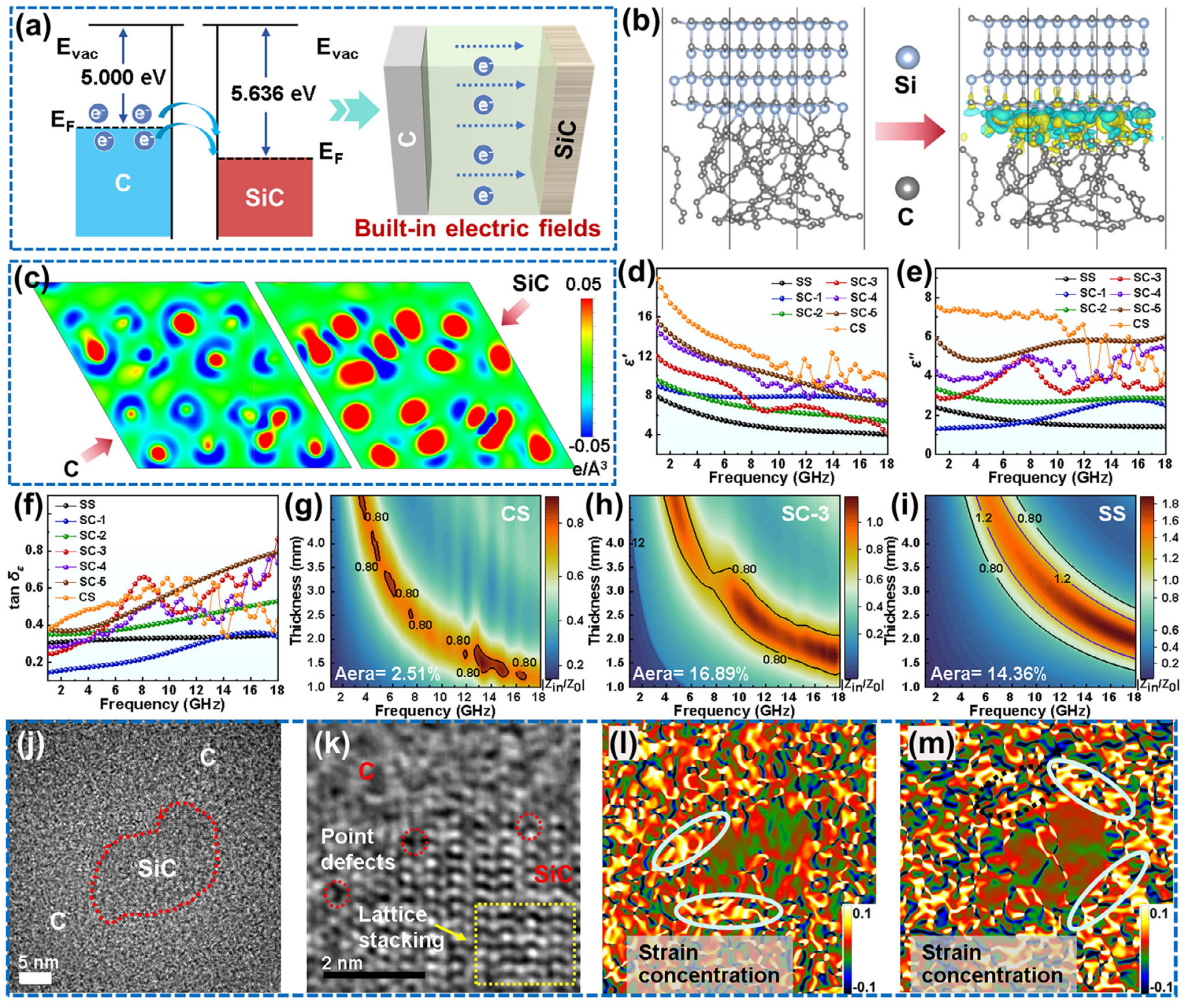
(a) Schematic illustration of electron transfer and the formation mechanism of the built‐in electric field at the heterogeneous interface between the C layer and the SiC layer; (b) Heterogeneous interface model of SiC and C together with the corresponding 3D differential charge density distribution (yellow indicates charge accumulation, and blue indicates charge depletion); (c) 2D cross‐sectional differential charge density map at the SiC and amorphous C interface (red represents charge accumulation, and blue represents charge depletion); (d) ε′, (e) ε″, and (f) tan δ_ε_ of the samples; 2D representations of |Z_in_/Z_0_| for (g) CS, (h) SC‐3, and (i) SS; (j, k) HRTEM images of SC‐3; Strain distribution maps along the ε_xx_ direction (l) and the ε_yy_ direction (m) in region j, where colors from green to dark blue indicate compressive strain and colors from red to bright yellow indicate tensile strain.

The electromagnetic parameters of all samples were further measured by VNA (Figure 3d–f). Owing to the high conductivity of CS, large values of ε′, ε″, and dielectric loss tangent (tan δ_ε_) are observed in the 2–8 GHz range, which tend to induce impedance mismatch and enhance electromagnetic reflection. After introducing the SiC component to form the Janus nanostructure, ε′, ε″, and tan δ_ε_ decrease significantly. Meanwhile, SC‐3 maintains a tan δ_ε_ above 0.5 in the 10–18 GHz range, indicating excellent dielectric loss capability, whereas SS exhibits relatively weak dielectric attenuation.

The influence of the Janus nano‐interface on impedance matching behavior was subsequently analyzed. As shown in Figure S21, the EMA performance of all samples follows the quarter‐wavelength matching condition [44]:

(1) $$t_{m}=\frac{n\lambda }{4}=\frac{\mathrm{nc}}{\left(4f_{m}{\left(\left|\mu_{r}\left|\;\right|\varepsilon_{r}\right|\right)}^{\frac{1}{2}}\right)},\left(n=1,\;3,\;5\text{…}\right)$$

where t_m_ and f_m_ denote the matching thickness and corresponding frequency, respectively. When this condition is satisfied, the phase difference between incident and reflected waves reaches 180°, resulting in destructive interference. The simulated and experimental t_m_ values agree well, and the λ/4 curves explain the thickness‐dependent RL behavior. Notably, SC‐3 achieves an accumulated EAB of 13.7 GHz across a thickness range of 1–5 mm, representing the overall frequency coverage obtained under different matching thicknesses. This broad cumulative bandwidth highlights the excellent thickness adaptability of SC‐3, enabling efficient microwave absorption over a wide range of practical operating conditions. Analysis of Figure S21a3–c3 shows that most RL_min_ values coincide with impedance matching regions where |Z_in_/Z_0_| approaches unity, indicating minimized reflection. As summarized in Figure 3g–i and Figure S22, the impedance‐matched area fraction (|Z_in_/Z_0_| = 0.8–1.2) of SS reaches 14.36%, much higher than that of CS (2.51%), suggesting superior impedance matching of pure SiC shells over pure carbon shells [45]. With increasing carbon shell thickness in the Janus SC series, the matched area first increases and then decreases, directly reflecting the intrinsic imbalance between attenuation capability and impedance matching in absorbers. SC‐3 exhibits the highest matched area fraction of 16.89%, 6.7 times that of CS and slightly higher than SS, demonstrating that the confined interfacial strategy markedly optimizes impedance matching and promotes microwave penetration into the interior for enhanced dissipation. Consistently, comparison of RL and |Z_in_/Z_0_| at a thickness of 2.54 mm (Figure S23) shows strong overlap between the EAB and impedance‐matched regions, further highlighting the critical role of impedance regulation.

HRTEM characterization of the SiC/C interface in SC‐3 (Figure 3j,k) reveals abundant point defects and lattice stacking near the SiC side adjacent to the carbon layer. Geometric phase analysis (GPA) of the HRTEM images (Figure 3l,m) directly maps the local strain distribution, showing pronounced tensile strain concentrated at the SiC/C interface. This originates from lattice mismatch induced by the carbothermal reduction‐driven transformation of amorphous carbon toward crystalline SiC under confined interfacial conditions, leading to interfacial stress concentration [46]. Defects and localized lattice strain break the periodicity of the crystal lattice, serving as charge‐trapping centers that induce nonuniform local electric fields and dipole formation. In an alternating electromagnetic field, these dipoles undergo orientation polarization, known as defect‐induced dipolar polarization, thereby promoting efficient electromagnetic energy dissipation [47].

The presence of defects and localized strain disrupts lattice periodicity and acts as charge trapping centers, generating nonuniform local electric fields and dipole formation. Under an alternating electromagnetic field, these dipoles undergo orientation polarization, namely defect‐induced dipolar polarization, which effectively enhances electromagnetic energy dissipation.

Electromagnetic response behaviors of CS, SS, and SC‐3 coatings were further simulated using CST Studio Suite, and the corresponding electric field distributions and power loss densities are presented in Figure 4a [48]. The CS coating exhibits a highly concentrated and strongest electric field at the surface region, which tends to induce strong reflection of incident electromagnetic waves. In contrast, the introduction of SiC reduces the surface electric field intensity and improves impedance matching. Moreover, SC‐3 shows the highest power loss density, followed by CS and SS. Therefore, SC‐3 maintains a moderate electric field distribution, avoiding impedance mismatch caused by excessive conductivity while ensuring sufficient penetration and effective attenuation of electromagnetic waves within the material. As illustrated in Figure 4b, the SiC/C interface is further constructed to simulate the microwave dissipation process. The simulations reveal relatively low power loss densities in the individual SiC and C domains, whereas the SiC/C interface displays markedly higher power loss, underscoring the dominant role of interfacial polarization. These findings align closely with the preceding electromagnetic parameter analysis, impedance matching behavior, and dielectric loss mechanisms, further confirming that the Janus interface enables synergistic optimization of impedance matching and energy dissipation, leading to enhanced EMA performance.

**FIGURE 4:**
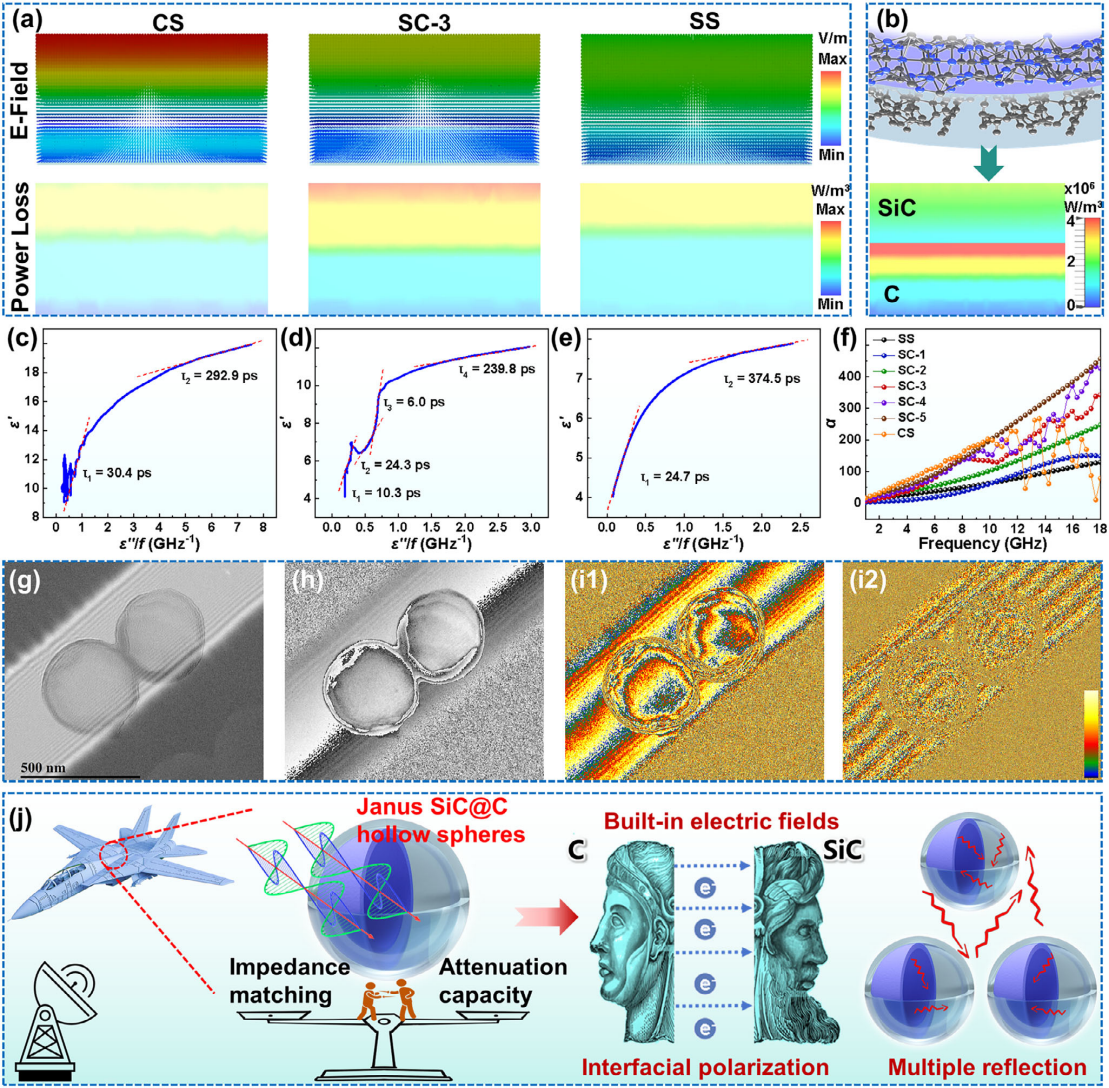
(a) Electric field intensity distribution and power loss density of the CS, SC‐3, and SS samples; (b) Schematic illustration of the SiC/C interface and the corresponding power loss density map; (c) SS, (d) SC‐3, and (e) SS ε′–ε″/f plots; (f) Attenuation constant (α) of the samples; (g) TEM image and (h) off‐axis electron holography of SC‐3, together with the corresponding (i) charge density distribution maps (i1–i2: progressively increasing electrical signal, where bright yellow indicates high charge density); (j) Schematic illustration of the EMA mechanism of the Janus SiC@C hollow nanospheres.

To further clarify the electromagnetic loss mechanism, ε″ vs. ε′ (Cole–Cole semicircle) plots were analyzed (Figure S24). According to Debye relaxation theory, the relationship between ε′ and ε″ is expressed as [49, 50]:

(2) $$\varepsilon^{\prime }=\varepsilon_{\infty }+\frac{\varepsilon_{s}-\varepsilon_{\infty }}{1+{\left(2\pi f\right)}^{2}\tau^{2}}$$

(3) $$\varepsilon^{\prime \prime }=\frac{2\pi f\tau \left(\varepsilon_{s}-\varepsilon_{\infty }\right)}{1+{\left(2\pi f\right)}^{2}\tau^{2}}$$

(4) $${\left(\varepsilon^{\prime }-\frac{\varepsilon_{s}-\varepsilon_{\infty }}{2}\right)}^{2}+{\left(\varepsilon^{\prime \prime }\right)}^{2}={\left(\frac{\varepsilon_{s}-\varepsilon_{\infty }}{2}\right)}^{2}$$

where ε_s_ and ε_∞_ are the static dielectric constant and high‐frequency limiting dielectric constant, respectively, and τ and ω (ω = 2πf) are the polarization relaxation time and angular frequency, respectively. When polarization relaxation occurs, the ε′–ε″ curve forms a semicircle, each corresponding to one Debye relaxation process. As shown in Figure 4f and Figure S24, the semicircles of the prepared materials are distorted and followed by a linear tail, indicating strong conductive loss. Comparison of semicircle numbers reveals that SC‐3 possesses more relaxation processes, which can be attributed to defect‐induced dipolar polarization and interfacial polarization generated by the built‐in electric field at the Janus interface.

For polarization‐dominated dielectric systems, ε′ typically shows a linear relationship with ε″/f, where the slope k = 1/(2πτ) represents the relaxation time [51]:

(5) $$\varepsilon^{\prime }=\frac{1}{2\pi \tau }\;\frac{\varepsilon^{\prime \prime }}{f}+\varepsilon_{\infty }$$

Fitting results (Figure 4c–e) indicate that CS and SS contain two linear regions corresponding to two relaxation times, whereas SC‐3 exhibits four distinct linear regions, suggesting richer polarization relaxation behavior. These findings confirm that the Janus nanostructure, constructed through confined interfacial engineering, significantly enhances polarization processes and improves dielectric loss capabilities. Furthermore, the attenuation constant (α) is a crucial parameter for evaluating a material's capability to dissipate microwaves, expressed as follows [52]:

(6) $$\alpha =\frac{\sqrt{2}\pi f}{c}\times \sqrt{\left(\mu^{\prime \prime }\varepsilon^{\prime \prime }-\mu^{\prime }\varepsilon^{\prime }\right)+\sqrt{{\left(\mu^{\prime \prime }\varepsilon^{\prime \prime }-\mu^{\prime }\varepsilon^{\prime }\right)}^{2}+{\left(\mu^{\prime }\varepsilon^{\prime \prime }+\mu^{\prime \prime }\varepsilon^{\prime }\right)}^{2}}}$$

Figure 4f reveals that CS exhibits the highest α in the 2–10 GHz range, whereas SC‐5 dominates at 10–18 GHz, reaching a maximum above 450, highlighting the strong attenuation of thick carbon shells but also the risk of severe impedance mismatch. In contrast, SC‐3 maintains a moderate α value below 300 across the entire frequency range while preserving relatively high attenuation capability in the 10–18 GHz region, indicating effective electromagnetic dissipation with balanced impedance matching. These results demonstrate that rational regulation of carbon shell thickness enables an optimized trade‐off between attenuation capability and impedance matching.

Off‐axis electron holography combined with TEM directly visualizes the Janus interface region of SC‐3 (Figure 4g–i), providing an effective approach to probe charge density distribution and dielectric loss characteristics [53]. TEM observation clearly reveals the SiC/C core‐shell interface. Under an alternating electromagnetic field, free carriers are captured by interfaces and defects during transport, leading to charge accumulation in localized regions. As shown in Figure 4h, the nonuniform carrier distribution near the heterointerface generates dipole moments and space polarization zones, inducing displacement polarization of the built‐in electric field, namely interfacial polarization. With increasing electromagnetic excitation (Figure 4i), charge accumulation becomes more pronounced, further strengthening interfacial polarization. These observations directly confirm that the Janus nano‐interface created by confined interfacial engineering induces strong polarization loss and enhances EMA performance.

Based on the above analyses, the EMA mechanism of SC hollow nanoshells is summarized in Figure 4j. First, SiC formed in situ under carbon‐shell‐confined diffusion, together with the hollow architecture, effectively enhances impedance matching. Second, the conductive carbon shell establishes efficient electron transport pathways, including migration and hopping, thereby reinforcing conductive loss under an external electromagnetic field. Third, multiple reflections and scattering within the hollow interior and between shells further promote electromagnetic energy dissipation. In addition, abundant defects in crystalline SiC capture carriers and induce defect‐related polarization. Most importantly, the large specific surface area of the nanoshell supplies abundant interfacial coupling sites, while the work function difference between SiC and carbon generates a built‐in electric field. Under an alternating electromagnetic field, strong polarization relaxation at the Janus interface dissipates electromagnetic energy, ultimately leading to outstanding EMA performance.

## 3 Conclusions

This study addresses the long‐standing challenge of achieving the synergistic optimization of impedance matching and electromagnetic attenuation in microwave‐absorbing materials. From the perspective of nanoscale asymmetric interfacial regulation, a class of structurally well‐defined SiC@C Janus hollow nanoshells was constructed, together with a corresponding confined interfacial engineering strategy. Owing to this unique architecture, outstanding electromagnetic protection performance was achieved at an ultralow filler loading of only 10 wt.%, including an RL_min_ of ‐62.36 dB, an EAB of 7.82 GHz, and an RCS reduction value of 24.91 dB m^2^. Multiscale analyses further reveal that the coupling between the nanoscale Janus asymmetric interface and the hollow structure is the key factor enabling efficient electromagnetic energy dissipation. The hollow Janus nanoshell markedly reduces the effective permittivity and optimizes impedance matching through the introduction of air and the low‐permittivity SiC phase. Meanwhile, the asymmetric C/SiC heterointerface and the internal hollow‐shell configuration effectively induce multiple reflections, scattering, and interfacial polarization losses, thereby enabling efficient electromagnetic energy dissipation while maintaining moderate conductive loss. The confined diffusion‐driven Janus nano‐interface construction strategy established in this work breaks through the conventional design paradigm of macroscale layered Janus structures, realizing a transition from macroscopic functional partitioning to precise nanoscale interfacial regulation. This provides a new perspective for understanding the intrinsic role of asymmetric interfaces in electromagnetic responses. Importantly, the proposed approach is not fundamentally restricted to a specific material system and thus exhibits excellent scalability, offering a general pathway for constructing structurally designable and performance‐tunable electromagnetic functional materials across diverse composite absorber systems.

## 4 Experimental Section

### 4.1 Raw Materials

Tetraethyl orthosilicate (TEOS, SiO_2_: 38.0–43.0%), ammonia solution (28 wt.%), and hydrofluoric acid (HF, 10 wt.%) were purchased from Shanghai Macklin Biochemical Co., Ltd. Absolute ethanol, resorcinol, formaldehyde, and deionized water were obtained from Sinopharm Chemical Reagent Co., Ltd. All reagents were of analytical grade and used as received without further purification.

### 4.2 Synthesis of SiO_2_@Phenolic Resin Nanospheres

First, 3 mL of TEOS and 4 mL of ammonia solution were added to a mixed solution containing 50 mL of absolute ethanol and 7 mL of deionized water. The mixture was magnetically stirred at room temperature for 6 h to allow complete hydrolysis of TEOS and formation of SiO_2_ nanospheres. Subsequently, 0.4 g of resorcinol and 0.456 g of formaldehyde were introduced into the above suspension and continuously stirred for 3 h, during which a phenolic resin layer was formed on the SiO_2_ surface via condensation polymerization. The obtained products were centrifuged (8000 rpm, 5 min), washed three times, and dried in a vacuum oven at 80°C for 12 h to yield SiO_2_@phenolic resin precursor powders, denoted as SiO_2_@PR‐1. Keeping TEOS and solvent conditions constant, the precursor composition was systematically tuned by varying resorcinol/formaldehyde amounts to 0.8 g/0.912 g, 1.2 g/1.368 g, 1.6 g/1.824 g, and 2.0 g/2.280 g, yielding SiO_2_@PR‐2, SiO_2_@PR‐3, SiO_2_@PR‐4, and SiO_2_@PR‐5, respectively. This rational gradient adjustment of phenolic resin content enables precise regulation of the coating growth behavior on SiO_2_ templates, thereby effectively tuning the evolution of the subsequent SiC@C Janus architecture. The final SC‐1 to SC‐5 products exhibit a continuous and well‐defined morphological evolution with increasing particle size, corresponding to the controlled formation of Janus hollow structures, as further confirmed by SEM analysis in Figures S5 and S6.

### 4.3 Synthesis of SiC@C Janus Hollow Nanospheres

The obtained precursors were placed in a tube furnace and heated to 650°C at a rate of 5°C min^−1^ under an argon atmosphere, followed by holding for 6 h to carbonize the phenolic resin into a carbon layer. The temperature was then further increased to 1400°C and maintained for 2 h, during which a carbothermal reduction reaction occurred between SiO_2_ and carbon, forming a SiO_2_@SiC@C sandwich structure. The high‐temperature products were subsequently immersed in 10 wt.% HF solution and ultrasonically etched for 24 h to selectively remove the residual SiO_2_ core. After washing and drying, SiC@C Janus hollow nanospheres were obtained and denoted as SC‐1, SC‐2, SC‐3, SC‐4, and SC‐5, corresponding to different precursor shell thicknesses. For comparison, SiO_2_@C obtained by carbonization at 650°C for 4 h was etched with HF to produce pure carbon hollow nanospheres (CS). In addition, pure SiC hollow nanospheres (SS) were obtained by calcining SC‐3 in air at 500°C for 2 h.

### 4.4 Characterization

The phase composition was identified by x‐ray diffraction (XRD, Bruker D8‐Discover, Cu Kα radiation, 40 kV). Raman spectra were collected using a confocal Raman microscope (Alpha300 Access, WITec GmbH). Fourier transform infrared spectroscopy (FTIR, Nicolet iS10, Thermo Fisher Scientific) was employed to analyze chemical structures. The microstructures were observed by field‐emission scanning electron microscopy (FE‐SEM, FEI Nova 450). Transmission electron microscopy (TEM, HRTEM, EDS mapping, and off‐axis electron holography) was performed using a JEM‐2100F field‐emission transmission electron microscope. Off‐axis electron holography was conducted under in situ electric‐field excitation to probe the electric‐field‐induced interfacial charge response and potential variation associated with the Janus heterointerfaces, providing insights into the polarization behavior underlying their electromagnetic response. Surface chemical compositions were determined by x‐ray photoelectron spectroscopy (XPS, Thermo ESCALAB 250XI). For electromagnetic measurements, the samples were homogeneously dispersed in paraffin and pressed into toroidal specimens (10 wt.% filler loading; outer diameter 7.00 mm, inner diameter 3.04 mm, thickness ≈2.00 mm). The electromagnetic parameters (complex permittivity and permeability) were measured using a vector network analyzer (Agilent N5244A). The reflection loss (RL) was calculated according to transmission‐line theory [54]:

(7) $$\mathrm{RL}=20\mathrm{lg}\left|\frac{Z_{\mathrm{in}}-Z_{0}}{Z_{\mathrm{in}}+Z_{0}}\right|$$

(8) $$Z_{in}=Z_{0}\;\sqrt{\mu_{r}/\varepsilon_{r}}\tan h\left[j\left(\frac{2\pi fd}{c}\right)\sqrt{\mu_{r}\varepsilon_{r}}\right]$$

where Z_in_, Z_0_, d, f, and c represent the input impedance of the absorber, impedance of free space, thickness of the absorber, frequency, and speed of light, respectively.

The radar cross section (RCS) in the far‐field response was simulated using CST Studio Suite with a metal‐backed configuration consisting of a 20 × 20 cm^2^ double‐layer square plate: a 2.54 mm absorbing layer atop a 1.0 mm PEC plate. A linearly polarized plane wave was incident along the +Z to −Z direction, with electric‐field polarization along the X‐axis. Open boundary conditions were applied in all directions, and the field monitor frequency was set to 11.88 GHz. The scattering direction was defined by spherical coordinates (θ, φ), and the RCS value was calculated as [55]:

(9) δ(dBm2)=10lg((4πS/λ2)|Es/Ei|)2

where S, λ, E_s_, and E_i_ represent the area of the target simulation model, wavelength of the electromagnetic wave, scattered wave's electric field intensity, and incident wave's electric field intensity, respectively. Details of first‐principles calculations and electromagnetic‐loss simulations are provided in the Supporting Information.

## Author Contributions

**Limeng Song**: Writing – original draft, Writing – review and editing, validation, methodology, investigation. **Cheng Song**: methodology, formal analysis, data curation. **Linan Wang**: investigation, methodology. **Li Guan**: conceptualization, methodology, investigation. **Yongqiang Chen**: investigation, methodology. **Duo Chen**: methodology, investigation. **Hailong Wang**: investigation, validation. **Bingbing Fan**: writing – review and editing, supervision, funding acquisition, investigation. **Shihao Jia**: investigation, formal analysis. **Yabo Huang**: investigation, validation. **Feiyue Hu**: writing – original draft, writing – review and editing, methodology, investigation, validation. **Huagao Wang**: methodology, investigation, validation. **Kesong Yu**: investigation. **Xin Xu**: investigation, formal analysis. **Qiancheng Gao**: funding acquisition, investigation. **Dou Zhang**: investigation. **Haijiao Xie**: software, investigation. **Rui Zhang**: writing – review and editing, investigation, methodology, supervision.

## Conflicts of Interest

The authors declare no conflicts of interest.

## Supporting information




**Supporting File**: advs77021‐sup‐0001‐SuppMat.docx.

## Data Availability

The data that support the findings of this study are available from the corresponding author upon reasonable request.
