# Supplementary material for: Janus Nanostructures Enabled by Confined Interfacial Engineering for Enhanced Electromagnetic Response
Source: Adv Sci (Weinh). 2026 Aug 6;13(60):e77021. doi: 10.1002/advs.77021 (PMC13445319; doi:10.1002/advs.77021)
Supplement: Supplementary file 1 [file ADVS-13-e77021-s001.docx]

**Janus nanostructures enabled by confined interfacial engineering for enhanced electromagnetic response**

Limeng Song^1, 2^, Cheng Song^1^, Feiyue Hu^3^^[1]^, Yongqiang Chen^2^, Linan Wang^4^, Huagao Wang^4^, Li Guan^1^, Duo Chen^1^, Kesong Yu^1^, Yabo Huang^1^, Shihao Jia^1^, Xin Xu^1^, Qiancheng Gao^1^, Dou Zhang^5^, Hailong Wang^2^, Haijiao Xie^6^, Rui Zhang^1, 2, 4*^, and Bingbing Fan^2, 4*^

1 School of Materials Science and Engineering, Zhengzhou University of Aeronautics, Zhengzhou 450015, P. R. China

2 School of Materials Science and Engineering, Zhengzhou University, Zhengzhou 450001, P. R. China

3 State Key Laboratory of Engineering Materials for Major Infrastructure, School of Materials Science and Engineering, Southeast University, Nanjing 211189, P. R. China

4 Institute of Advanced Ceramics, Henan Academy of Sciences, Zhengzhou 450046, P. R. China

5 Department of Astronautical Science and Mechanics, Harbin Institute of Technology, Harbin, 150001, P. R. China

6 Hangzhou Yanqu Information Technology Co., Ltd, Hangzhou 310003, P. R. China

1. **First principles calculation details：**

In model construction, bulk structure of amorphous C150 was constructed by *Ab-initio* Molecular Dynamics (AIMD) method. In AIMD simulation, bulk structure of amorphous C150 was annealed to 5000 K in 5000 fs. Then C150 was bathed in NVT ensemble in 1000 fs using Nosé-Hoover thermostat. After bathing in NVT ensemble, a superlattice of C150 bulk structure was constructed based on amorphous C150. Finally, the bulk structure was sliced along (001) to obtain slab model of amorphousC(001). Slab model of SiC(111) 2×2×1 supercell with four stoichiometric layers was constructed based on SiC conventional standard cell (JCPDS #29-1129). Slab model of SiC(111)/amorphousC(001) was constructed based on SiC(111) 2×2×1 and amorphous C(001). In slab model, thickness along c direction was set at 35Å to avoid weak interactions between images.

In Density Functional Theory (DFT) calculations, structural optimizations were performed by Vienna *Ab-initio* Simulation Package (VASP) with the projector augmented wave (PAW) method. The exchange-functional was treated using the Perdew-Burke-Ernzerhof (PBE) functional, in combination with the DFT-D3 correction. Cut-off energy of the plane-wave basis was set as 450 eV. For optimization of lattice size of SiC bulk structure, the Brillouin Zone integration was performed with a Monkhorst-Pack *k*-point sampling of 6×6×6. For optimization of lattice size of amorphous C150 bulk structure, the Brillouin Zone integration was performed with a *k*-point sampling of 2×2×3. For optimization of slab models, the the Brillouin Zone integration was performed with a *k*-point sampling of 2×2×1. The self-consistent calculations applied a convergence energy threshold of 10^-5^ eV. The equilibrium geometries and lattice constants were optimized with maximum stress on each atom within 0.02 eV Å^-1^. Spin polarization method was used in structural optimization to describe magnetism of models. Isosurface level of charge density difference was set at 0.03 e Å^-3^. Saturation level of 2D charge density difference was set from -0.05 to 0.05. Work function of slab model were calculated by GGA-PBE functional, and then treated by Vaspkit interface.

2. **Electromagnetic simulation details:**

The intrinsic electromagnetic responses of the CS, SC-3, and SS samples were investigated using the Unit Cell module in CST Studio Suite. A simplified rectangular stealth-coating model with a lateral size of 10 × 10 mm^2^ and a thickness of 2.54 mm was constructed to analyze the electric-field distribution and power-loss density, thereby providing deeper insight into the EMA mechanism. An incident microwave with a frequency of 11.88 GHz was excited from the top of the model through a waveguide port and propagated along the z-axis. The boundary conditions were defined as follows: electric boundary along the x-direction (Et = 0), magnetic boundary along the y-direction (Ht = 0), and open boundary along the z-direction. The electromagnetic parameters used in the simulations were derived from the experimentally measured values obtained by the vector network analyzer. Furthermore, a simplified SiC/C Janus interfacial model was established, in which the upper layer consisted of SiC and the lower layer of amorphous carbon, to evaluate the influence of the Janus interface on the electromagnetic power loss density.

3. **Supplementary figures and tables:**

Figure S1. XRD patterns for these samples.

Figure S2. FTIR spectra for these samples.

Figure S3. Raman spectra for these samples.

Figure S4. (a) XPS survey spectrum, (b) Si 2p spectrum, (c) C 1s spectrum, and (d) O 1s spectrum for the SC-3 sample.

Figure S5. SEM images: (a1) SiO_2_@phenolic resin-1, (b1) SiO_2_@SiC@C-1, and (c1) SC-1 sample; (a2) SiO_2_@phenolic resin-2, (b2) SiO_2_@SiC@C-2, and (c2) SC-2 sample.

Figure S6. SEM images: (a1) SiO_2_@phenolic resin-4, (b1) SiO_2_@SiC@C-4, and (c1) SC-4 sample; (a2) SiO_2_@phenolic resin-5, (b2) SiO_2_@SiC@C-5, and (c2) SC-5 sample.

Figure S7. SEM images of CS.

Figure S8. SEM images of SS.

Figure S9. EDS mapping of the SC-3 sample.

Figure S10. HRTEM image of the SC-3 sample, where (a) is the SiC layer and (b) is the corresponding SAED pattern.

Figure S11. (a) 3D plots and (b) 2D representations of RL for SC-1.

Figure S12. (a) 3D plots and (b) 2D representations of RL for SC-2.

Figure S13. (a) 3D plots and (b) 2D representations of RL for SC-4.

Figure S14. (a) 3D plots and (b) 2D representations of RL for SC-5.

Figure S15. 2D comparison of the EAB for all samples.

Figure S16. 3D RCS plot of the (a) PEC and PEC coated with (b) CS and (c) SS.

Figure S17. 3D RCS plot of the PEC coated with (a) SC-1, (b) SC-2, (c) SC-4, and (d) SC-5.

Figure S18. 2D RCS plots for all samples.

Figure S19. Work function of SiC and amorphous C.

Figure S20. 1D differential charge density at the SiC/C interface.

Figure S21. 2D plots of the RL, the relationship between thickness and peak frequency (quarter-wavelength mode), and impedance matching rate (|Z_in_/Z_0_|) at different thickness of (a) CS, (b) SC-3, and (c) SS, respectively.

Figure S22. 2D representations of |Z_in_/Z_0_| at different thickness of (a) SC-1, (b) SC-2, (c) SC-4, and (d) SC-5.

Figure S23. 2D plots of |Z_in_/Z_0_| and RL at 2.54 mm of SC-3

Figure S24.Cole-Cole semicircles of (a) CS, (b) SS, (c) SC-1, (d) SC-2, (e) SC-3, (f) SC-4, and (g) SC-5.

**Table S1.** **EMA properties of comparative absorbers.**

| Materials | Fill rate (wt.%) | RL_min_ (dB) | d_1-RLmin_ (mm) | \|RL_min_/d_1_\| (dB/mm) | EAB (GHz) | d_2-EAB_ (mm) | EAB/d_2_ (GHz/mm) | Ref. |
| --- | --- | --- | --- | --- | --- | --- | --- | --- |
| 2D-Co@C | 20 | -22.87 | 1.82 | 12.56 | 6.41 | 1.82 | 3.53 | [1] |
| Co@TiO_2_ | 20 | -47.35 | 2.5 | 18.94 | 6.32 | 2.5 | 2.53 | [2] |
| 2D Fe_3_C | 50 | -52.09 | 2.90 | 17.96 | 2.55 | 1.20 | 2.12 | [3] |
| SnS/SnS_2_/SnO_2_/CF | 50 | -46.74 | 2.0 | 23.37 | 5.28 | 1.7 | 3.10 | [4] |
| CoNiMn@C | 40 | -30.1 | 2.0 | 15.05 | 5.8 | 2.0 | 2.9 | [5] |
| RE-MoS_2_ | 60 | -52.02 | 2.6 | 20.00 | 7.12 | 2.6 | 2.74 | [6] |
| PAN/C NFs | 30 | -44.73 | 1.76 | 25.41 | 6.60 | 2.07 | 3.19 | [7] |
| MXene/C | 15 | -53.02 | 3.8 | 13.95 | 5.3 | 2.4 | 2.21 | [8] |
| SiC NFs@CF | 50 | -44.04 | 1.2 | 36.7 | 3.44 | 1.1 | 3.13 | [9] |
| FeS/MoS_2_@NC | 30 | -53.01 | 1.63 | 32.52 | 5.20 | 1.72 | 3.02 | [10] |
| SiC/SiO_2_@TiO_2_/SnO_2_ | 100 | -66.2 | 1.98 | 33.43 | 3.4 | 1.98 | 1.72 | [11] |
| SiBCN/SiC_nw_ aerogel | 20 | -50.1 | 2.0 | 25.05 | 6.4 | 2.0 | 3.2 | [12] |
| **SC-3** | **10** | **-62.36** | **2.54** | **25.55** | **7.82** | **2.54** | **3.07** | **Here** |

**Reference**

[1]	Y. Liu, J. Zhou, C. Li, H. Zhang, Y. Wang, Y. Yan, L. Duan, Z. Cheng, Y. Ma, Z. Yao, *Nature Communications* **2025**, *16*, 202.

[2]	Z. Gao, A. Iqbal, T. Hassan, S. Hui, H. Wu, C. M. Koo, *Advanced Materials* **2024**, *36*, 2311411.

[3]	R. Zhao, T. Gao, Y. Li, Z. Sun, Z. Zhang, L. Ji, C. Hu, X. Liu, Z. Zhang, X. Zhang, G. Qin, *Nature Communications* **2024**, *15*, 1497.

[4]	Z. Guo, D. Lan, Z. Jia, Z. Gao, X. Shi, M. He, H. Guo, G. Wu, P. Yin, *Nano-Micro Letters* **2025**, *17*, 23.

[5]	M. Huang, B. Li, Y. Qian, L. Wang, H. Zhang, C. Yang, L. Rao, G. Zhou, C. Liang, R. Che, *Nano-Micro Letters* **2024**, *16*, 245.

[6]	J. Wen, S. Hui, Q. Chang, G. Chen, L. Zhang, X. Fan, K. Tao, H. Wu, *Advanced Functional Materials* **2024**, *34*, 2410447.

[7]	J. Xiao, B. Zhan, M. He, X. Qi, X. Gong, J. L. Yang, Y. Qu, J. Ding, W. Zhong, J. Gu, *Advanced Functional Materials* **2025**, *35*, 2316722.

[8]	F. Wu, P. Hu, F. Hu, Z. Tian, J. Tang, P. Zhang, L. Pan, M. W. Barsoum, L. Cai, Z. Sun, *Nano-Micro Letters* **2023**, *15*, 194.

[9]	W. Geng, Y. Liu, H. Lei, L. Song, H. Wang, G. Shao, Y. Zhu, R. Zhang, Z. Min, B. Fan, *Chemical Engineering Journal* **2024**, *499*, 155785.

[10]	Y. Shen, Z. Ma, F. Yan, C. Zhu, X. Zhang, Y. Chen, *Advanced Functional Materials* **2025**, *35*, 2423947.

[11]	H. Zhang, G. Deng, X. Sun, L. Yan, G. Han, Z. Cui, Y. Zhang, Y. Zhang, K. Xie, R. Yu, J. Shui, X. Liu, *Advanced Functional Materials* **2025**, *36*, e24866.

[12]	J. Jiang, L. Yan, Y. Xue, J. Li, C. Zhang, X. Hu, A. Guo, H. Du, J. Liu, *Chemical Engineering Journal* **2024**, *482*, 148878.

1. Corresponding authors.

   E-mail: hufeiyue@seu.edu.cn (F. Hu), zhangray@zzu.edu.cn (R. Zhang), fanbingbing@zzu.edu.cn (B. Fan).
